# Surgical resection for patients with recurrent or metastatic gastrointestinal stromal tumors: a protocol for a systematic review and meta-analysis update

**DOI:** 10.1186/s13643-021-01863-7

**Published:** 2021-12-06

**Authors:** Zhaolun Cai, Chunyu Xin, Zhiyuan Jiang, Chunyu Liu, Chaoyong Shen, Guozhen Zhao, Yuan Yin, Xiaonan Yin, Zhou Zhao, Mingchun Mu, Bo Zhang

**Affiliations:** 1 Department of Gastrointestinal Surgery, West China Hospital, Sichuan University, Chengdu, 610041 Sichuan China; 2 Institute for International Health Professions Education and Research, China Medical University, Shenyang, 110122 Liaoning China; 3 Department of Pharmacy, West China Second University Hospital, Sichuan University, Chengdu, 610041 Sichuan China; 4 Evidence-Based Pharmacy Center, West China Second University Hospital, Sichuan University, Chengdu, 610041 Sichuan China; 5 Key Laboratory of Birth Defects and Related Diseases of Women and Children (Sichuan University), Ministry of Education, Chengdu, 610041 Sichuan China; 6 West China School of Pharmacy, Sichuan University, Chengdu, 610041 Sichuan China; 7 Beijing Institute of Traditional Chinese Medicine/Beijing Hospital of Traditional Chinese Medicine, Beijing, 100010 China; 8 Sanya People’s Hospital/West China (Sanya) Hospital, Sichuan University, Sanya, 572022 China

**Keywords:** Gastrointestinal stromal tumor, Metastatic, Recurrent, Surgery, Tyrosine kinase inhibitor

## Abstract

**Background:**

There are limited data on the clinical benefits of adding surgical resection in patients with recurrent or metastatic gastrointestinal stromal tumors (GISTs). This protocol outlines the planned scope and methods for a systematic review and meta-analysis update that will compare the clinical outcomes of surgical resection combined with tyrosine kinase inhibitor (TKI) with TKI treatment alone in patients with recurrent or metastatic GISTs.

**Methods:**

This review will update a previously published systematic review by our team. This protocol is presented in accordance with the PRISMA-P guideline. PubMed, Embase, and Cochrane Central Register of Controlled Trials will be systematically searched and supplemented by a secondary screening of the references of all included studies. We will include randomized controlled trials (RCTs) and non-randomized studies (NRS) in this review update. The outcomes evaluated will be overall survival and progression-free survival. Two reviewers will independently screen and select studies, extract data from the included studies, and assess the risk of bias of the included studies. Data extracted from RCTs and NRS will be analysed and reported separately. Preplanned subgroup analyses and sensitivity analyses are detailed within this protocol. The strength of the body of evidence will be assessed using GRADE.

**Discussion:**

This systematic review and meta-analysis update will provide a current assessment of the evidence for the role of surgery in patients with recurrent or metastatic advanced GISTs. These findings will be used by the Chinese Society of Clinical Oncology (CSCO) GIST guideline recommendations on surgical treatment for recurrent or metastatic advanced GIST patients in China.

**Systematic review registration:**

This protocol was prospectively registered in the Open Science Framework Registry (https://osf.io/xus7m).

**Supplementary Information:**

The online version contains supplementary material available at 10.1186/s13643-021-01863-7.

## Introduction

Gastrointestinal stromal tumors (GISTs) are the most common mesenchymal tumors of the gastrointestinal tract. GISTs mostly have an activating mutation in the gene encoding the KIT proto-oncogene, receptor tyrosine kinase (KIT) or platelet-derived growth factor receptor alpha (PDGFRα) receptor tyrosine kinase [1, 2]. Therefore, KIT and PDGFRα-directed tyrosine kinase inhibitors (TKIs) are widely used for advanced GISTs with activated KIT or PDGFRα in vitro. However, a complete pathological response is rare [3], and progression and/or secondary resistance inevitably occurs with long-term treatment of advanced disease [4, 5]. First-line treatment with imatinib for patients with advanced GISTs results in response or tumor control in more than 80% of patients. However, nearly 50% of patients with advanced GISTs have progressive disease within two years [6]. Thus, various treatment strategies including surgery combined with TKIs, resumption of imatinib (IM), IM dose escalation or other targeting agents have been investigated to improve the survival of patients with recurrent or metastatic GISTs [7–12].

To summarize conflicting evidence comparing surgery combined with TKIs and TKIs alone in treating recurrent or metastatic GISTs, our team performed a systematic review and meta-analysis and demonstrated that surgery combined with TKIs therapy is associated with better overall survival (OS) and progression-free survival (PFS) [13]. However, the total sample size was not very large, and the certainty of evidence for both outcomes was not assessed by the Grading of Recommendations Assessment Development and Evaluations (GRADE) approach.

Over the past few years, the results of several studies concerning this topic have been published, potentially providing evidence of the effects of surgery combined with TKIs for people with recurrent or metastatic GISTs. Given the ongoing uncertainty regarding the benefits of surgery combined with TKIs, the primary objective of this systematic review is to update the previous study by identifying and incorporating recent research data to evaluate the survival benefits of surgery combined with TKIs for patients with recurrent or metastatic GISTs across all clinical settings, and the findings will be used by the Chinese Society of Clinical Oncology (CSCO) GIST guideline recommendations.

## Methods

This review will update a previously published systematic review and meta-analysis by our team [13].

### Protocol and registration

The present protocol has been registered at the Open Science Framework Registry (https://osf.io/xus7m). This protocol is presented in accordance with the Preferred Reporting Items for Systematic Reviews and Meta-Analysis Protocols (PRISMA-P) guidelines (see PRISMA-P checklist in Additional file 1, [14]). This updated systematic review and meta-analysis will be conducted according to the PRISMA 2020 statement and the standard methodology recommended by the Cochrane Collaboration [15–17].

### Inclusion criteria

The inclusion criteria complied with the PICOS (population, intervention, comparators, outcomes and study design) description model [18] to detail the main elements. There will be no restrictions on language or publication year. The criteria that studies will be considered for inclusion are as follows:

- Population: Patients with recurrent and/or metastatic gastrointestinal stromal tumors according to the histologic examination. The study population will include all patients, with no restrictions based on country, race and ethnic origin, age, sex, or job.
- Intervention: Surgical intervention combined with TKI. Radiofrequency ablation is also regarded as a surgical intervention.
- Comparators: TKI therapy alone.
- Outcomes and measurement:

#### Primary outcome

- OS: death from any cause.

#### Secondary outcome

- PFS: progression, distant recurrence, or death from any cause.

OS and PFS are time-to-event outcomes. Thus, the hazard ratio (HR) will be used to pool overall effects [17]. HR a common assessment reported in epidemiologic studies, is defined as the hazard within the exposed groups divided by the hazard within the unexposed groups to calculate the effect of the treatment [19].

- Study design: Eligible study designs will include non-randomized studies (NRS) (case–control studies, and cohort studies) and randomized controlled trials (RCTs).
- Other inclusion criteria: Studies with a minimum of 60 months of follow-up reporting survival (time to event) outcomes. In addition, the studies need to provide sufficient data to calculate or estimate HRs and 95% confidence intervals (CIs).

### Exclusion criteria

- Studies with overlapping data.
- Conference abstracts, letters, case reports, reviews or nonclinical studies without available data will be excluded.
- There are missing or insufficient data after a reasonable attempt at contacting the corresponding authors.
- Full-text articles are not available after exhaustive searches to locate the texts.

### Information sources and search strategy

A systematic search of PubMed, Embase (Ovid) and the Cochrane Central Register of Controlled Trials databases will be performed from their inceptions to 30^th^ December 2021 to identify all relevant studies. The details of the PubMed database search strategy are shown in Additional File 2. We will manually screen the references of all included articles to further identify additional studies meeting the eligibility criteria, and their full texts will be retrieved.

### Study selection and data extraction

All study records collected in the literature search will be imported to EndNote software. After removing duplicates, two authors will independently screen all articles identified from the database search based on the eligibility criteria outlined above. First, the two independent reviewers will screen titles and abstracts. Subsequently, the two independent reviewers will reassess the full texts of the identified studies, verifying the reasons for inclusion and exclusion. The screening process will be shown in a PRISMA flow diagram [20].

Data extraction for the included studies will be conducted by two authors independently using a standardized electronic data extraction form that was piloted by all reviewers. If multiple studies are conducted on the same subjects, only the study with the highest methodological quality, the most complete results, or the most recent published date will be included [21].

All disputes in the process of study selection and data extraction will be resolved through team discussion.

### Dealing with missing data

When a study does not report HR and its 95% CI, we will contact the corresponding author of this study to request missing data via email. If no effective response is received in 14 days, we will try to estimate some or all the lnHR, the log-rank observed minus expected events (O-E), the log-rank variance and the variance of the lnHR by indirect methods [22]. If even these indirect methods cannot be applied, we will estimate HRs based on Kaplan-Meier curves [22].

### Risk of bias assessment

Two independent reviewers will assess the methodological quality/risk of bias of the included studies, and disagreements will be resolved by team discussion [23]. RCTs will be assessed for risk of bias with Cochrane Collaboration’s tool [24]. NRS will be assessed with the Risk of Bias In Non-randomised Studies of Interventions (ROBINS-I) tool [25].

### Data analysis

If identified as possible (the studies retrieved have quantitative data reported that can be combined), the extracted data will be aggregated into a meta-analysis by STATA version 15.0 (STATA, College Station, TX) software. HRs and 95% CIs will be pooled to measure time-to-event outcomes in consideration of the number and timing of events. Statistical significance will be defined as p < 0.05. HRs derived from the multivariate analysis will be used as default. When multivariate values are absent, univariate values will be used. Data from RCTs and NRS will be analysed and reported separately.

Chi-square-based Q-test will be used to check heterogeneity. The I^2^ test will be used to quantify the effect of heterogeneity. For chi-squared values with p < 0.1, heterogeneity will be considered to be significantly high. I^2^ with values 0% to 40% represents not important, 30% to 60% moderate, and 50% to 90% substantial heterogeneity, respectively [17]. Random effects models will be used for meta-analysis in cases of significant heterogeneity (I^2^ > = 50% or p < 0.1). Any comparison with high heterogeneity will be explored by subgroup analyses or sensitivity analysis. In addition, the study design and characteristics in the included studies will be analysed.

### A priori subgroup analyses

If multiple studies with homogenous outcomes are reported within the following subgroups, planned subgroup analyses of the primary outcome include the following:

1. Classification of recurrent or metastatic GISTs: initially metastatic GIST versus recurrent GIST.
2. Response to preoperative TKI therapy: complete response/partial response or stable disease versus progressive disease.
3. Surgical intervention following TKI therapy: yes versus not.
4. TKI at time of surgery: imatinib treatment versus multiple lines of TKIs treatment.
5. HR extracted from: multivariate analysis versus univariate analysis.
6. NRS with propensity score analyses : yes versus not.

### Sensitivity analysis

To evaluate whether the results of the meta-analysis are substantially influenced by the presence of any individual study, we will conduct a sensitivity analysis by omitting studies with a high risk of bias.

### Meta-biases and quality of evidence

If over ten studies are available, funnel plot symmetry will be used to examine publication bias [17]. We will use the GRADE approach to assess the quality of findings systematically [26], which is considered an effective method to provide detailed information on assessments [27]. The quality of findings will be classified as high, moderate, low, and very low according to four dimensions: risk of bias, inconsistency, indirectness, and imprecision. High-quality findings will indicate a high grade of confidence in intervention efficacy and quality. The GRADE assessments will be presented in a summary table.

## Discussion

Our previous work was published three years ago and has some limitations [13]. Over the past years, several studies focusing on this topic have been published. This systematic review and meta-analysis update will provide a current assessment of the evidence for the role of surgery in patients with recurrent or metastatic advanced GISTs. The findings from this review will build the foundation for future research and highlight the implications for clinical practice, and the results will be used by the CSCO GIST guideline to help develop recommendations on the recurrent or metastatic disease in China.

## Amendments

The protocol for the study will be amended if required. Any protocol amendments will be updated in the Open Science Framework Preregistration and explicitly described in the final manuscript.

## Supplementary Information

**Additional file 1.**

**Additional file 2.**

## Data Availability

Not applicable.

## Abbreviations

CIs: Confidence intervals
CSCO: Chinese society of clinical oncology
GRADE: Grading of Recommendations Assessment Development and Evaluations
GISTs: Gastrointestinal stromal tumors
HR: Hazard ratio
IM: Imatinib
KIT: KIT proto-oncogene, receptor tyrosine kinase
NRS: Non-randomized studies
OS: Overall survival
PDGFRα: Platelet-derived growth factor receptor alpha
PFS: Progression-free survival
PRISMA: Preferred Reporting Items for Systematic Reviews and Meta-Analyses
PRISMA-P: Preferred Reporting Items for Systematic Reviews and Meta-Analysis Protocols
RCTs: Randomized controlled trials
TKIs: Tyrosine kinase inhibitors
