# Supplementary material for: Surgical resection for patients with recurrent or metastatic gastrointestinal stromal tumors: a protocol for a systematic review and meta-analysis update
Source: Syst Rev. 2021 Dec 6;10:306. doi: 10.1186/s13643-021-01863-7 (PMC8650510; doi:10.1186/s13643-021-01863-7)
Supplement: Supplementary file 2 — Additional file 2. [file 13643_2021_1863_MOESM2_ESM.docx]

Additional File 2. Search strategy for PubMed.

| 1 | (("gastrointestinal stromal tumors"[MeSH Terms] OR ("gastrointestinal"[All Fields] AND "stromal"[All Fields] AND "tumors"[All Fields]) OR "gastrointestinal stromal tumor*"[All Fields]) OR ("GIST")) OR ("GISTS") |
| --- | --- |
| 2 | "recurrance"[All Fields] OR "recurrence"[MeSH Terms] OR "recurrence"[All Fields] OR "recurrences"[All Fields] OR "recurrencies"[All Fields] OR "recurrency"[All Fields] OR "recurrent"[All Fields] OR "recurrently"[All Fields] OR "recurrents"[All Fields] OR ("metastatically"[All Fields] OR "metastatics"[All Fields] OR "metastatization"[All Fields] OR "metastatize"[All Fields] OR "metastatized"[All Fields] OR "metastatizing"[All Fields] OR "secondary"[MeSH Subheading] OR "secondary"[All Fields] OR "metastatic"[All Fields]) OR ("disease progression"[MeSH Terms] OR ("disease"[All Fields] AND "progression"[All Fields]) OR "disease progression"[All Fields] OR "progression"[All Fields] OR "progress"[All Fields] OR "progressed"[All Fields] OR "progresses"[All Fields] OR "progressing"[All Fields] OR "progressions"[All Fields] OR "progressive"[All Fields] OR "progressively"[All Fields] OR "progressives"[All Fields]) |
| 3 | 1 AND 2 |
| 4 | "surgery"[Subheading] OR "surgery"[All Fields] OR "surgical procedures, operative"[MeSH Terms] OR ("surgical"[All Fields] AND "procedures"[All Fields] AND "operative"[All Fields]) OR "operative surgical procedures"[All Fields] OR "general surgery"[MeSH Terms] OR ("general"[All Fields] AND "surgery"[All Fields]) OR "general surgery"[All Fields] OR "surgery's"[All Fields] OR "surgerys"[All Fields] OR "surgeries"[All Fields] |
| 5 | "metastasectomy"[MeSH Terms] OR "metastasectomy"[All Fields] OR "metastasectomies"[All Fields] |
| 6 | "resect"[All Fields] OR "resectability"[All Fields] OR "resectable"[All Fields] OR "resectates"[All Fields] OR "resected"[All Fields] OR "resecting"[All Fields] OR "resection"[All Fields] OR "resectional"[All Fields] OR "resectioned"[All Fields] OR "resectioning"[All Fields] OR "resections"[All Fields] OR "resective"[All Fields] OR "resects"[All Fields] |
| 7 | 4 OR 5 OR 6 |
| 8 | 3 AND 7 |
| 9 | animals [mh] NOT humans [mh] |
| 10 | (a case[Title]) |
| 11 | case report*[Title] |
| 12 | case series[Title] |
| 13 | review[Title] |
| 14 | meta-analysis[Title/Abstract] |
| 15 | 8 NOT 9 NOT 10 NOT 11 NOT 12 NOT 13 NOT 14 |
